# Preparing to deliver a stepped wedge cluster-randomised trial to test the effectiveness of daily symptom tracking integrated into electronic health records for managing rheumatoid arthritis: a mixed-methods feasibility trial

**DOI:** 10.1186/s41927-025-00464-4

**Published:** 2025-02-17

**Authors:** Katie L. Druce, Yumna Masood, Helen Chadwick, Sarah Skyrme, Deb Griffiths-Jones, Ramiro D. Bravo Santisteban, Peter Bower, Jill Firth, Charlotte A. Sharp, Christopher J. Armitage, Dawn Dowding, John McBeth, Caroline Sanders, William G. Dixon, Sabine N. van der Veer, on behalf of the REMORA team

**Affiliations:** 1 Centre for Epidemiology Versus Arthritis, Division of Musculoskeletal and Dermatological Sciences, Faculty of Biology Medicine and Health, The University of Manchester, Manchester Academic Health Science Centre, Manchester, UK; 2 Centre for Health Informatics, Division of Informatics, Imaging and Data Science, Faculty of Biology, Medicine and Health, The University of Manchester, Manchester Academic Health Science Centre, Vaughan House, Portsmouth Street, Manchester, M13 9GB UK; 3 Core Research Facilities, Technology Platforms, Faculty of Biology, Medicine and Health, The University of Manchester, Manchester, UK; 4 NIHR ARC Greater Manchester, Centre for Primary Care and Health Services Research, Faculty of Biology, Medicine and Health, The University of Manchester, Manchester Academic Health Science Centre, Manchester, UK; 5 Pennine MSK Partnership, Integrated Care Centre, Oldham, UK; 6 Division of Musculoskeletal and Dermatological Sciences, The University of Manchester, Manchester Academic Health Science Centre, Manchester, UK; 7 Kellgren Centre for Rheumatology, Manchester Royal Infirmary, Manchester University NHS Foundation Trust, Manchester, UK; 8 Manchester Centre for Health Psychology, Division of Psychology and Mental Health, The University of Manchester, Manchester Academic Health Science Centre, Manchester, UK; 9 Division of Nursing, Midwifery and Social Work, School of Health Sciences, Faculty of Biomedicine and Health, The University of Manchester, Manchester Academic Health Science Centre, Manchester, UK; 10 Division of Population Health, Heath Services Research and Primary Care, The University of Manchester, Manchester Academic Health Science Centre, Manchester, UK; 11 Rheumatology department, Salford Royal Hospital, Northern Care Alliance NHS Foundation Trust, Salford, UK

**Keywords:** Rheumatoid arthritis, Feasibility studies, Patient-generated health data, Smartphone applications, mHealth, Symptoms

## Abstract

**Background:**

We sought to assess the feasibility of a stepped-wedge cluster-randomised trial testing the effectiveness of a complex mHealth intervention called REMORA: a co-designed smartphone app enabling daily, weekly and monthly symptom tracking integrated into electronic health records for people with rheumatoid arthritis (RA).

**Methods:**

We conducted a mixed-methods feasibility trial using a convergent approach with some explanatory sequential elements. Patients were eligible to take part if they were older than ≥18 years of age, had (suspected) RA or undifferentiated inflammatory arthritis, and consented to take part from two outpatient departments. We analysed quantitative app and electronic health record data descriptively. We analysed qualitative data from interviews and clinic observations thematically. We assessed four feasibility domains: recruitment and consent (target: 15 patients per site), intervention uptake (≥70% of recruited participants completed on-boarding, i.e., registered with the app and submitted at least one symptom report), intervention adherence (>50% daily symptom reports provided), and measuring disease activity as the primary outcome (scores available for ≥80% of people with a follow-up clinic visit). Due to time constraints, we only recruited patients to the intervention group, leaving us unable to test the logistics of randomising sites in accordance with the trial’s cluster stepped wedge design.

**Results:**

Of 130 people screened, 52 consented. Of those, 32 (62%) completed on-boarding. On-boarded participants provided symptom data on 2384/3771 (63%) of possible days. Among the 48 people who had ≥1 follow-up appointment, at least one disease activity scored was obtained for 46 (96%) of them. Factors related to intervention uptake formed the biggest threat to trial feasibility, including lack of clarity of communication and guidance, access to technology, and personal challenges (e.g., being busy or unwell).

**Conclusion:**

We found that delivering a trial to test the effectiveness of integrated symptom tracking in rheumatology outpatient settings was feasible. The future REMORA trial will contribute to the much-needed evidence base for the impact of integrated symptom tracking on care delivery and patient outcomes, including decision-making, patient experience, disease activity, and symptom burden.

**Trial registration:**

This feasibility trial was registered at https://www.isrctn.com/ on 23-Jan-2023 (ISRCTN21226438).

**Supplementary information:**

The online version contains supplementary material available at 10.1186/s41927-025-00464-4.

## Introduction

Rheumatoid arthritis (RA) is an exemplar for long-term conditions that may benefit from remote monitoring, with data integrated into health information systems and clinical workflows [1]. People living with RA, a common immune-mediated inflammatory disease, typically receive outpatient rheumatology care 1–4 times a year, with fluctuations in well-being and symptoms, such as pain and fatigue, between visits [2–4]. Recall and descriptions of these fluctuations are poor, hampering optimal clinical and self-management [2, 4, 5]. Developments in mobile technology and health apps have revolutionised possibilities for clinical- and self-management of long-term conditions, including rheumatic diseases (such as RA), by minimising the reliance on patient recall through frequent symptom tracking and therefore providing a clearer and more accurate picture of changing symptoms through time [2, 6].

Up to 86% of people with rheumatic diseases are willing to use symptom monitoring apps to improve their disease management [7, 8], and healthcare professionals responsible for treating RA perceive a benefit from gaining insight into the day-to-day lived experience of their patients [9]. Evidence from a range of long-term conditions has indicated that the collection of patient-generated health data and its integration into electronic health records (EHRs) could improve shared decision-making and patients’ satisfaction and self-management, and decrease anxiety [4, 8, 10–12]. Further potential benefits may include more efficient utilisation of healthcare services, benefiting not just patients, but service providers and the wider economy [13].

Despite growing interest in integrating these complex remote monitoring interventions into clinical systems and processes, evidence of their impact on services and outcomes remains scarce [14]. Previous studies were not randomised, small (e.g., single centre studies), recruited a highly selected sample, collected symptoms infrequently (weekly/monthly), used low-tech interventions (e.g., SMS), and/or did not integrate the tracked symptom data in EHRs [14–18].

We previously demonstrated proof-of-concept of the REmote MOnitoring of Rheumatoid Arthritis (REMORA) system, a complex mHealth intervention that enables people living with RA to track their symptoms daily, integrate REMORA data into the EHR and share these with their rheumatology team [6]. Having shown that both patients and clinicians were positive about the intervention, we wished to scale up its use and study its impact on clinical outcomes using a multi-centre stepped wedge cluster randomised trial; in the remainder of the manuscript, we refer to this trial as the “REMORA trial” [19]. The trial aims to evaluate the effectiveness of the integrated symptom tracking intervention on care delivery and patient outcomes, such as disease activity, decision-making, patient experience, and other patient priorities, such as pain and fatigue.

Prior to undertaking the REMORA trial, we needed to gain a comprehensive understanding of the likely feasibility of, and potential barriers to, conducting such a trial, to understand whether it would be feasible to proceed, and to optimise recruitment and participation. Therefore, the current study aimed to better understand the feasibility of our proposed trial by assessing rates of recruitment and consent, intervention uptake, intervention adherence and primary outcome completion, and exploring the factors that influenced these rates.

## Methods

### Context: a planned cluster-randomised stepped wedge trial to evaluate the effectiveness of integrated symptom tracking (the REMORA trial)

The REMORA trial formed the context for the current feasibility trial and guided its design. The REMORA trial will be conducted within 16 rheumatology outpatient departments (i.e., sites) in England, United Kingdom (UK), using randomisation at site-level (i.e., cluster randomisation). Randomisation will follow a stepped wedge design, i.e., randomisation determines the time at which sites switch over from recruiting participants to standard-of-care to integrated symptom tracking. This means that patients who are recruited after a site’s switch-over will be allocated to using the integrated symptom tracking intervention (see Figure S1 for a visualisation of the trial design). Follow-up will last 12 months from date of recruitment for each participant, with clinical evaluation based on routine visits requested by clinical care teams, rather than additional research visits. The primary outcome measure will be disease activity as recorded by clinical care teams; disease activity score for 28 joints (DAS-28) for in person appointments and clinical disease activity index (CDAI) for remote appointments [20]. Secondary outcomes, collected via web surveys, include patient reported symptoms (e.g. pain, fatigue), work productivity and disease activity (e.g. joint counts, patient global). A mixed-methods process evaluation will determine the effectiveness, and underlying mechanisms, of the intervention. We refer to the REMORA trial protocol for further details [19].

### Feasibility setting, participants, intervention, and procedures

#### Design and setting

We reported the current feasibility trial in accordance with the CONSORT 2010 statement extended for pilot and feasibility trials [21] and the consolidated criteria for reporting qualitative research (COREQ) [22] (Tables S1 and S2 in the supplementary material).

We conducted an integrated mixed-method feasibility trial [23, 24] that complemented assessment of traditional quantitative feasibility performance measures (such as rates of recruitment and intervention uptake) with qualitative data on participants’ experiences and suggestions for overcoming barriers to successful trial delivery. We used a convergent approach (i.e., concurrent data collection and analysis) with some explanatory sequential elements (i.e., quantitative data analysis guided some of the qualitative data collection and analysis) [25]. The study took place in two rheumatology outpatient departments in Greater Manchester, United Kingdom. Although we had originally planned to test the logistics of sites switching over from recruiting patients to standard-of-care to recruiting them to the intervention group, time constraints meant we were unable to assess this aspect as part of the feasibility trial.

#### Participants

We first recruited and consented rheumatology healthcare professionals responsible for patient care to take part in the study. This included consent for reviewing the symptom tracking data in consultations for consented patients with an optional interview and/or clinic observations. Site recruitment teams then identified potential patient participants under the care of consented healthcare professionals. Eligible patients were adults (i.e. ≥18 years of age) with confirmed or suspected RA or undifferentiated inflammatory arthritis, and an Android or iOS smartphone with daily internet access. They were asked to report daily, weekly, and monthly symptoms tracking using the REMORA app (see ‘Intervention’ below for more detail). No restrictions were placed on the level of disease activity experienced by patients at the point of consent. As the REMORA app was only available in English, we excluded patients who could not speak and understand English and had no support from someone who did.

Potential participants were given a participant information sheet (including a section on ‘What is the purpose of the research’) and time to review the study information and ask questions, before providing informed consent to take part in the feasibility trial. We also asked consent to be contacted for an additional interview and/or consultation observation. Individuals who declined participation in the feasibility trial were asked consent for being interviewed about their reason(s) for not wanting to take part; we did not keep a record of how many and why people refused to be interviewed or observed. Written or verbal consent before interviews and/or observations was obtained.

#### Intervention

The REMORA system is a complex mobile health (mHealth) intervention comprising a co-designed smartphone app that enables people living with RA to track their symptoms daily, weekly and monthly. The app is linked to regional data infrastructure for integrating symptom data into participating local hospitals’ EHR systems; this facilitates review of the data at forthcoming outpatient consultations. REMORA has been co-designed with members of our patient and public involvement and engagement (PPIE) group, who have been instrumental in developing and refining the app and supporting materials. The REMORA system was well received by patients and healthcare professionals in an initial proof-of-value study at a single site and showed potential to enhance clinical encounters [26].

REMORA users provided daily reports for the seven symptoms from the Rheumatoid Arthritis Impact of Disease (RAID) score [27] on 0–10 visual analogue scales (VAS): pain, function, fatigue, sleep, physical well-being, emotional well-being and coping; they also reported their duration of morning stiffness on a 7-point ordinal scale. They were also asked to submit weekly and monthly questionnaires on domains such as self-reported flares, work productivity [28], and disability [29] (supplementary figure S2 shows screenshots of the REMORA app).

This patient-generated symptom data was automatically sent daily to a secure server managed by the regional Integrated Care Board (i.e., the body responsible for regional healthcare service delivery). Data was then presented graphically via a bespoke, interactive REMORA dashboard available within the local EHR system using single sign-on. This meant that when a healthcare professional logged onto a particular patient’s record, they had immediate access to that patient’s symptom data without the need to sign-on again or searching for the patient. Healthcare professionals received training for accessing and using the dashboard to support them reviewing the symptom data, discussing this with the patient during their consultation, and making treatment decisions accordingly. Symptom data was not routinely reviewed in between visits and patients were advised to use normal procedures for seeking help in the event of flare or difficulty. Data was only visible to healthcare professionals who had undergone training and been provided with access (see supplementary figure S3 for a screenshot of the interactive dashboard).

#### Trial procedures under evaluation

We evaluated the feasibility of the REMORA trial’s design and processes across four feasibility domains: (1) Recruitment and consent, (2) Intervention uptake: “On-boarding”, (3) Intervention adherence: “Completeness of symptom tracking”, and (4) Outcome measurement. We specified *a-priori* criteria to assess each domain (Table 1). These criteria were informed by our previous proof-of-concept study [6], discussions with the research team and our PPIE group, and peer-reviewed as part of applying for external funding for the REMORA trial.

**Table 1:** A priori assessment criteria used to evaluate the feasibility domains in this feasibility study

Domain	Assessment criteria	Trial feasible	Trial feasible with adjustments	Trial not feasible
(1) Recruitment and consent	Number of patients per site consented to symptom tracking	≥15	11–14	≤10 per site
(2) Intervention uptake: “On-boarding”	Proportion of consented participants who successfully downloaded and registered with the app and completed at least one symptom report	≥70%	50–69%	<50%
(3) Intervention adherence: “Completeness of symptom tracking”	Proportion of study days on which participants recorded at least one symptom out of all possible days	>50%	25–50%	<25%
(4) Outcome measurement	Proportion of participants who had ≥1 follow-up visit and at least one disease activity score available	≥80%	50–79%	<50%

##### Recruitment and consent

Sites were asked to recruit as many members as possible of the rheumatology teams primarily responsible for making treatment decisions, to maximise available patient participants and for ease of follow up by teams. Sites were then each asked to recruit up to 30 eligible patients over a period of 13 weeks, with a minimum target of 5 per month (i.e., the 15 participants per site required to meet the “Trial feasible” threshold). This sample size allowed us to estimate a participant follow-up rate of 80% to within a 95% confidence interval of ±14%.

##### Intervention uptake: “On-boarding”

**Fig. 1:**
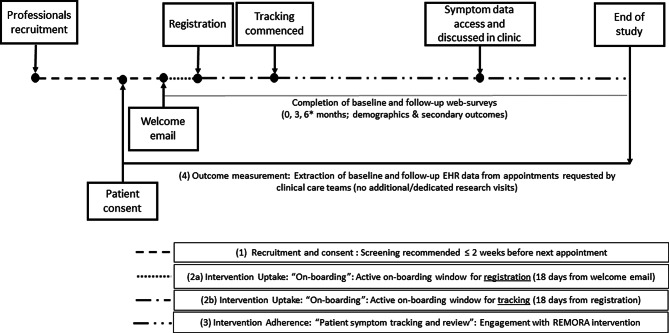
Study design for the feasibility trial. *Although data collection was intended to run for up to 6 months, no participants achieved 6 months follow-up, so no requests to complete the 6-month web survey were sent

Following consent, trial participants received a welcome email with instructions to download, register with and use the REMORA app (Fig. 1). The welcome email included a link to a baseline web survey for collecting additional demographic data and secondary outcome measures, including work productivity, disability and resource use. Participants were considered ‘on-boarded’ if they appeared in app registration logs as having successfully submitted their unique study identifier and activation code, completed permissions to link and create/use the NHS login, and submitted at least one symptom report. An active on-boarding window sought to encourage on-boarding within 18 days of the initial email being sent. Non-registration reminders were sent 3, 7 and 14 days after the initial invite, as required, via email. ‘Non-tracking’ reminders were sent to participants who had registered in the app successfully but had not recorded any symptoms within 3, 7 and 14 days of registration. Participants who had not completed on-boarding by day 18 were considered to have failed on-boarding. Registration after day 18 was possible but was not actively encouraged by further reminders.

##### Intervention adherence: “Completeness of symptom tracking”

An in-app notification prompted participants each day to complete their daily (at 6.30 pm, confirmed as a feasible time in our proof-of-concept study [6]), weekly (at 3.30 pm) and monthly (at 12.00 pm) questionnaires. Participants were followed for up to 6 months. Adherence to symptom tracking was calculated based only on the completion of the daily questionnaires. Additional reminders to symptom track were sent on up to two occasions if people had not completed at least one symptom per day on at least 50% of days between tracking commencement and days 7 and 14. In addition to this formal domain criteria, we explored adherence against a-priori defined adherence clusters of low (<25% days), moderate (25–60% days) and high adherence(>60% days).

##### Outcome measurement

Site staff extracted data from EHRs at baseline and for all follow-up visits that occurred in the follow-up window (up to 6 months). Data included demographics at baseline, clinical history, and disease activity. Collection of disease activity by clinicians during the clinical consultation is the primary outcome of the main trial, via the DAS28 for in-person appointments or CDAI for remote appointments, and thus successful completion of these metrics was the focus for our outcome measurement evaluation.

### Data collection

#### Demographics

Baseline demographic data were primarily collected from the EHR by site staff using a secure study-specific electronic data capture tool hosted at the research team’s institution. Extracted data included participants’ sex, date of birth (to calculate age), ethnicity, smoking status, Body Mass Index, recorded diagnosis (RA, suspected RA, undifferentiated inflammatory arthritis) and date of diagnosis (to calculate disease duration). Missing data for ethnicity and smoking status were replaced with data from a self-report web survey completed by participants at baseline.

#### Feasibility evaluation

The data for evaluating the feasibility of our proposed trial design came from a range of sources (Table 2). Briefly, quantitative data was obtained from sites, the app or via EHR extractions pertaining to screening and recruitment, app registration and daily symptom reports, and availability of disease activity scores. Healthcare professionals were asked to record how REMORA data were used within their consultation and whether they found it useful.

**Table 2:** Data sources used to explore the feasibility domains: (1) Recruitment and consent, (2) Intervention Uptake: “On-boarding”, (3) Intervention Adherence: “Completeness of symptom tracking”, (4) Outcome measurement

Data source	Description	Feasibility Domains			
		(1)	(2)	(3)	(4)
Screening/recruitment logs	Screening and recruitment logs were provided by sites, detailing the number and outcome of patient approaches, and reasons for ineligibility or declining participation.	X			
Quantitative data on registration rates (via app records)	Registration logs (including study identifier and date of registration) were obtained from the app records and used to calculate the rate of registration.		X		
Quantitative data on adherence rates (via app records)	App records provided information about all symptoms reported by participants each day, linked to their study identifier. Participants were considered to have “engaged” with the app on a day on which they provided at least one of the 8 daily symptom reports (see ‘Intervention’ description for more detail).			X	
Quantitative data on availability of disease activity at baseline/follow-up (via EHR extraction)	A comprehensive selection of data was extracted from the participants’ EHRs and inputted into a secure study-specific database. Of interest here are disease activity data only.				X
Healthcare professional reported symptom data use (questionnaire)	A brief survey recorded healthcare professionals use of the REMORA data within their consultation, including information about when they looked at the data, whether they looked at the data with the patient and how useful the data were. A free-text box was provided for any additional comments regarding how data were used during the consultation.			X	
Interviews with patients or clinicians	One-to-one semi-structured interviews (duration 7–35 min) took place by telephone, face-to-face, or using video-conferencing software, depending on participant preference. Discussion topics included the use of technology for health monitoring in general and, where applicable: 1. perceptions of the REMORA system 2. the impact of REMORA system and tracked symptom data on the clinical consultation and decision making 3. reasons for their (lack of) adherence with symptom tracking 4. reasons for declining participation in the study, or for not completing on-boarding	X	X	X	
Observations	Observations (duration 12 to 36 min) of consultations.			X	X
Study team logs	Study team logs comprised field notes from interviews, observations and summaries of contact/correspondence with participants or site staff via phone or email.	X	X	X	

Two researchers (YM and SS) conducted interviews via phone, video call or in person (depending on interviewees’ preferences) and observations in clinic using a pilot-tested topic guide. Both researchers were female, had PhDs, worked as post-doctoral research associates at the University of Manchester (UK), had significant experience in qualitative research, and had completed good clinical practice training. No relationship was established between the researchers and participants prior to data collection taking place. Interviews were conducted among (1) patients who declined symptom tracking participation (“decliners”), (2) those who consented to study participation, but did not register with the app by day 18 (“non-registered participants”), (3) those who registered with the app, stratified based on their level of adherence (see ‘Quantitative data analysis’ below for definitions), and (4) healthcare professionals. Participants were only interviewed once. A series of professional-patient dyads were also observed during consultations, where both parties had provided optional consent for their consultations to be observed. We observed up to a maximum of one and three consultations per patient and professional, respectively. Study team logs were maintained throughout the trial to detail field notes from interviews/observations and summaries of contact (phone/email) between the study team and participants or site staff.

### Data analysis

#### Quantitative data analysis

We evaluated study performance descriptively against the assessment criteria presented in Table 1, by determining rates of recruitment, on-boarding, adherence (based on the proportion of possible days on which participants tracked their symptoms) and completion of the primary outcome measure. We also explored adherence in terms of membership of one of the a-priori defined adherence clusters (see ‘Intervention adherence: Completeness of symptom tracking’ above), though this did not formally contribute to our feasibility criteria (1) and (2).

#### Qualitative data analysis

Interviews and observations were audio-recorded, transcribed, deidentified and thematically analysed using [30] NVivo 12 Plus software. The Theoretical Domains Framework guided our analysis of data from patients who tracked their symptoms by providing a lens for considering how the intervention influenced participants’ behaviours [31, 32]. For analysing the healthcare professional interviews and clinic observations, the Three Talk model of shared decision-making served as a guide [33] to examine the collaborative decision-making process between patients and professionals. The two researchers (YM and SS) systematically collated preliminary codes into potential themes using a constant comparative method, with review sessions with the wider research team to ensure that data extracts effectively represented analytic themes and to identify further subthemes where these emerged from the data. We did not share transcripts or findings with participants for feedback.

Study team logs were reviewed to complement the quantitative and qualitative data analyses by providing additional insight and contextual information.

#### Mixed-methods interpretation

Following guidance by Aschbrenner et al. [25], we created joint displays to bring together findings from the quantitative and qualitative analyses and interpret them together using an integrative approach.

## Results

### Recruitment and consent

**Fig. 2:**
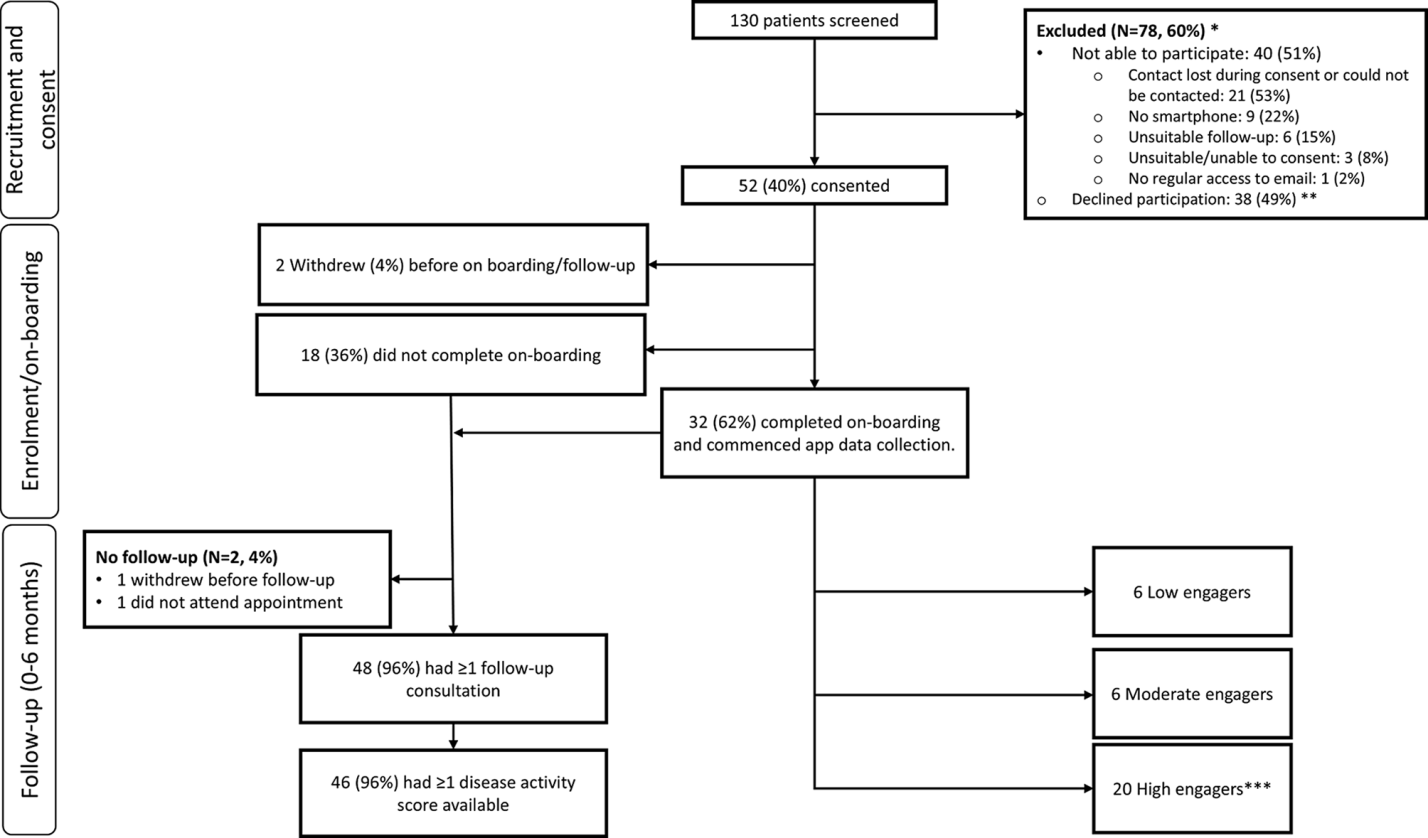
Flowchart of the REMORA2 feasibility trial domains (1) Recruitment and consent, (2) Intervention Uptake: “On-boarding” and (3) Intervention Adherence: “Completeness of symptom tracking”. Low adherers—symptoms reported on <25% days; Moderate adherers—symptoms reported on 25–60% days; High adherers—symptoms reported on >60% days. *We were unable to collect data on people who were excluded after screening as we did not have their consent to do this; **Some reasons for declining participation, explored during ‘decliner interviews’ can be found in Table 4; ***Includes one participant who withdrew after 9 days but was classified as a high engager while in the study

Across both sites, a total of 130 patients were screened for participation, of whom 78 (60%) were excluded as they either declined participation (n = 38, 49%) or were otherwise unable to take part (n = 40, 51%). For the latter group, the most common reason for not being consented was that we lost contact or could not reach people during the consent process (n = 21, 53%), which is a common logistic challenge in trials. This was followed by a lack of access to appropriate technology (smartphone/email; n = 10, 25%), an inclusion criterion that research nurses could not assess from information in the EHR when screening. Other reasons are shown in Fig. 2. In total 52 people were consented (20 at site 1 and 32 at site 2), which returned a “Trial feasible” evaluation for Domain (1).

### Intervention uptake: “On-boarding”

Among the 52 people recruited and consented (site 1: 32, site 2: 20), two (4%) withdrew before commencing on-boarding and 18 (36%) did not complete on-boarding, with the remaining 32 (62%) successfully completing on-boarding (see Table 3). Thus, we returned a “Trial feasible with adjustments” evaluation for Domain (2). To support on-boarding we sent participants a total of 76 email reminders. Of those 76, 66 reminders were sent for non-registration to 37 participants, of whom 17 (46%) then completed registration. Table S3 in the supplementary materials shows no substantial differences between those who were consented and those who on-boarded, though more people of white ethnicity appeared in the on-boarded group (94% (95%CI: 79–99) vs 87% (74–94), respectively).

### Intervention adherence: “Completeness of symptom tracking”

Among the 32 on-boarded participants, participants provided symptom data on 2384/3771 (62%) of possible days. Twenty (62%) participants achieved high adherence (see Table 3), while the low and moderate groups each comprised six (19%) participants. Nine reminders were sent to seven participants for having less than 50% of days since tracking, with two of them having a final completion rate of 78% and 97%. We observed few differences between low versus high engagers, though low engagers (n = 6) were younger (median (interquartile range) years: 42 (35–55)) than the high engagers (n = 20; 61 (51–65) and had a shorter disease duration (years: 1 (0–6) vs 3 (1–11) (Table S3 in supplementary material). Given that adherence rates exceeded the >50% threshold, we achieved a “Trial feasible” evaluation for Domain (3).

**Table 3:** Comparisons of demographic characteristics for a) those who were recruited versus those who on-boarded and b) low versus high adherers^1^

		Recruited vs on-boarded participants		Low vs High adherers^1^	
		Recruited (n = 52)	On-boarded (n = 32)	Low (n = 6)	High (n = 20)
Female (n, (%))		36 (69%)	23 (72%)	6 (100%)	13 (65.0%)
Age (median (IQR))		58 (48–65)	57 (48–63)	42 (35–55)	61 (51–65)
White ethnicity (n, (%))		45 (87%)	30 (94%)	4 (67%)	20 (100%)
Smoking^2^ (n, (%))	Current	11 (21%)	5 (16%)	1 (17%)	3 (15%)
	Former	22 (42%)	13 (41%)	2 (33%)	9 (45%)
	Never	18 (34%)	13 (41%)	3 (50%)	8 (40%)
BMI^3^ (n, (%))	Normal	17 (33%)	11 (34%)	2 (33%)	5 (25%)
	Over	19 (36%)	10 (32%)	1 (17%)	9 (45%)
	Obese	16 (31%)	11 (34%)	3 (50%)	6 (30%)
Diagnosis^4^ (n, (%))	RA	50 (96%)	31 (97%)	6 (100%)	20 (100%)
	Suspected RA	1 (2%)	1 (3%)	0	0
	UA	1 (2%)	0	0	0
Disease duration (median (IQR))		3 (0–10)	4 (0–11)	1 (0–6)	3 (1–11)

*Abbreviations CI* confidence interval, *IQR* interquartile range, *RA* rheumatoid arthritis, *UA* Undifferentiated inflammatory arthritis

^1^Low adherers—symptoms reported on <25% days; High adherers—symptoms reported on >60% days

^2^smoking status missing from medical record for one on-boarded participant

^3^No one is underweight, so category omitted

^4^No one suspected IA, so category omitted

### Outcome measurement

**Fig. 3:**
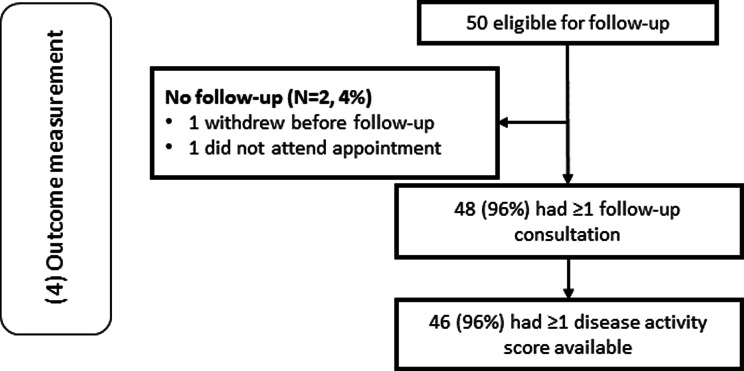
Flowchart of the REMORA2 feasibility trial domain (4) Outcome measurement

Figure 3 shows that, among 50 consented participants who did not withdraw before on-boarding, 49 were eligible to have a follow-up visit during the study window as one participant withdrew after nine days in the study, prior to follow-up. Of those 49, 48 (98%) had ≥1 appointment, of whom 46 (96%) had ≥1 disease activity score completed by a clinician in the consultation. Compared to the upper threshold of ≥80%, we therefore achieved a “Trial feasible” evaluation for Domain (4).

### Overall evaluation of feasibility domains

In summary, when comparing our quantitative findings to the assessment criteria outlined in Table 1, we confirmed that the REMORA trial was feasible for domains (1), (3) and (4). However, we found on-boarding rates were suboptimal, suggesting that domain (2) (intervention uptake) required adjustments to enhance its feasibility.

In total, we interviewed 28 patients, ranging from 23–80 years old and mostly women identifying as white and British. In addition, we interviewed seven healthcare professionals and observed five consultations. Interviews and observations lasted 7–35 and 12–36 min, respectively. Analysis of our qualitative data sources suggested thematic saturation and identified five challenges which differentially impacted on all four of the feasibility domains and should therefore be considered for modification in the main trial. The challenges which affected domain (2) will be discussed in detail below, with all other challenges being summarised and illustrated with quotes (where available) in Table 4.

#### Clarity of communication and guidance

Site staff and patient participants brought up issues around the clarity of communication and guidance. For example, some patient participants reported problems with downloading or using the REMORA app due to a lack of communication from the study team or unclear/complex guidance. It was specifically highlighted that the duration of the active on-boarding window was unclear, leaving some participants unsure if they could still register after a delay.

The study materials did not include a comprehensive privacy statement, which may explain why some patients who declined participation raised concerns about privacy and data security (e.g., safety of personal data). Although some patients who consented but did not on-board shared these concerns, many stated they were not concerned about this. This suggests this primarily affected recruitment, and to a lesser extent on-boarding.

#### Accessibility

Issues of accessibility were defined as any issues impacting only specific groups of patients, such as those who were older, less technologically literate, or more unwell. Accessibility issues had an extensive impact on the on-boarding rates, with some more modifiable than others.

With regards to modifiable accessibility issues, difficulties were experienced among consented participants whose device did not meet the specific requirements (e.g., an older phone running an out-of-date operating system, lack of a PIN code on the device to prevent unauthorized individuals from accessing tracked symptom data). Not all participants were aware of these requirements at the time of recruitment, leading to some being consented without then being able to on-board.

A second substantial challenge was that many participants indicated that they only checked emails irregularly or not at all, meaning that communications from the research team were often missed and therefore not actioned. For some participants, irregularly or not checking emails was normal, while others mentioned that this deviated from their normal email-checking behaviour because they had been busier than normal or had other competing priorities. Regardless, not reading the study instructions sent via email meant patient participants could not on-board.

Some participants also perceived issues surrounding the provision of on-boarding support: those who were more nervous about their ability to participate suggested they would have benefitted from greater support, while others had sought additional support from outside of the study team (e.g. from friends and family, or in clinic). Although participants could request support via email, some found it problematic that no telephone contact information was provided.

Patient participants felt that it could be difficult to integrate the study activities into their everyday life if they were busy, ill, or otherwise committed during the active on-boarding window. It was observed that the short active on-boarding window did not appropriately account for such events. Additional challenges included personal motivations and perceived benefits. Despite having consented to take part, several patients who did not on-board queried the specific benefits of the data collected within the app and its use in clinic, while others had not engaged with health apps in general or felt overwhelmed by how many were available.

Other, less modifiable personal barriers to participation included RA-related challenges (e.g., high levels of pain, dexterity, forgetfulness or brain fog) and non-RA related challenges (e.g., dyslexia, non-fluency in English, a lack of confidence with using technology).

## Discussion

Our mixed-methods feasibility trial demonstrated that our plans to evaluate REMORA within a multi-centre trial would be feasible with respect to recruitment, adherence, and outcome measurement. However, we also found that we require modifications to optimise intervention uptake, as many consented participants struggled to on-board successfully. The most common barriers identified in relation to this included a lack of clarity of communication and guidance relating to downloading and using the app, as well as accessibility issues (e.g., technical and personal challenges).

### Comparison to previous studies

In our study, the principal threat to the success of our proposed trial was intervention uptake. Previous studies showed that the limited uptake of smartphone apps among specific groups of people negatively affect their access, use and benefits of mHealth solutions [34]. For example, older people and those with lower socioeconomic backgrounds may be more likely to lack the necessary equipment [35] than their younger or more affluent counterparts. This may be a particular concern among those with RA, where prevalence and prognosis are associated with both age and deprivation [36]. In our study, we found that just over one-fifth of screened but excluded participants could not take part because they did not have access to a smartphone at all, or had one that was not compatible with the REMORA app. Furthermore, irregular checking emails and a lack of familiarity with apps in general were reported as barriers to intervention uptake. While we could not link these health equity issues directly to individuals’ demographics, it is likely that it included older and more socially deprived patients within our target population.

Another digital determinant of health is digital literacy [37], here referring to an individual’s ability to find, create and/or use health related information on or from electronic platforms. Greater digital literacy is associated with a higher belief in the usefulness of solutions such as health apps [38], which tends to be lower among older populations [39, 40]. In this study, several interviewed patients identified themselves as feeling too nervous to use the REMORA app, or as otherwise lacking adequate skills to participate without further support from the study team. In keeping with other studies [8, 39, 41], our findings suggested potential benefits from providing more structured guidance and tailored education for patients and varying methods of contact with the research team (e.g., email, SMS, phone calls). Evidence also indicates that healthcare professionals’ recommendations influence patients’ decisions to adopt an apps [41]. However, others have found that rheumatologists may be reluctant to use apps such as REMORA due to concerns that mHealth-based symptom monitoring may increase their workload [42]. We note too that in this study both patients and professionals expressed interest in using the app data during consultations but some perceived disinterest from the other party. This suggested that further training and support may be needed to enable integrated symptom tracking to be used effectively as part of shared decision-making during consultations.

### Study limitations

One limitation of our study was that, due to a lack of data on the date and approximate time of appointments, it was not possible to triangulate patients’ and professionals’ perceived use of the symptom data with actual use of this data in clinic as recorded by the interactive REMORA dashboard. We have therefore modified our data collection approach to mitigate this in the REMORA trial. This will now include the date and (approximate) time of the consultation when patients are seen, interactive REMORA dashboard data access logs, and information on both the patients’ and healthcare professionals’ perceived data use.

A second limitation was that, because of time constraints and logistical challenges, we did not recruit patients to a standard-of-care group, despite this being the case in the main trial to serve as the comparator group. This leaves it unknown if, and how, not receiving the intervention may affect recruitment rates and outcome measurements, as well as whether there may be operational challenges in sites switching over from recruiting to standard-of-care to recruiting to integrated symptom tracking. Mitigations to try and alleviate concerns regarding this include blinding site staff involved in recruitment to the time of switch-over, and extensive and continued engagement with participants as well as those involved in delivering the REMORA trial.

Lastly, a lack of translated versions of the REMORA app into other languages meant we could not recruit individuals who did not read English and had no-one who could help with this. Acknowledging this limitation, we conducted a separate piece of work to understand barriers to participation among those who do not receive their healthcare in English; a manuscript reporting the findings of this work is currently in preparation.

### Implications for trial design and conduct

This feasibility trial was designed to inform the delivery of a multi-centre stepped wedge cluster randomised trial to evaluate the effectiveness of integrated symptom tracking on disease activity and patient-reported outcomes, such as pain, fatigue and mood. We designed the REMORA trial to overcome a number of key methodological challenges from previous studies, such as small sample sizes, non-randomisation and use of low-tech interventions (e.g. web platforms, SMS services) [14–18, 43–46]. Our findings show that it is feasible to overcome these limitations and contribute the much-needed evidence to determine the effectiveness of similar digital health interventions to improve the care and outcomes of people with RA and other long-term conditions.

Based on the findings from the current feasibility trial, we made several modifications to the design of the REMORA trial, which is reflected in our trial protocol [19]. Key modifications include:

1. Clarification of our inclusion criteria, including the technical requirements of devices, to streamline recruitment of eligible participants;
2. Procedures and materials to ensure potential participants are more fully informed about what is required to initiate and maintain symptom tracking; these include co-produced patient information documentation; the development of video-based instructions, and provision of demonstration apps to site recruitment teams;
3. Extending the active on-boarding window for people to join the study, to better reflect that individuals may experience delays in ability to on-board including competing interests or the experience of ill-health, and diversify the method of reminders provided to include telephone calls by the research team;
4. Expanding the ways in which people can obtain support, including the use of a telephone, as well as email, helpdesk and peer support offered by our PPIE group. To further mitigate any issues with individuals infrequently accessing emails, we have also incorporated the use of text messages to alert participants to new email contacts, including welcome and reminder emails.

### Conclusions

This study demonstrated that it would be feasible to conduct a trial to test the effectiveness of REMORA, a co-designed smartphone app with integration of tracked symptom data into electronic health records. We have shown that several challenges impacted on the availability and use of technologies for mobile health studies and intervention uptake. These findings ensured that we are equipped to provide optimised support to enhance the success of the trial and the implementation of the intervention being tested. We believe that the REMORA trial will contribute robust evidence to determine the impact of integrated symptom tracking on key care and disease outcomes among individuals with long-term conditions such as RA.

## Electronic supplementary material

Below is the link to the electronic supplementary material.

Supplementary Material 1

## Data Availability

Requests for access to data and other study materials should be made in writing to the corresponding author.
