# Supplementary material for: Preparing to deliver a stepped wedge cluster-randomised trial to test the effectiveness of daily symptom tracking integrated into electronic health records for managing rheumatoid arthritis: a mixed-methods feasibility trial
Source: BMC Rheumatol. 2025 Feb 17;9:17. doi: 10.1186/s41927-025-00464-4 (PMC11834673; doi:10.1186/s41927-025-00464-4)
Supplement: Supplementary file 1 — Supplementary Material 1 [file 41927_2025_464_MOESM1_ESM.pdf]

Supplementary materials

Figure S1 – Stepped wedge cluster randomized controlled trial design

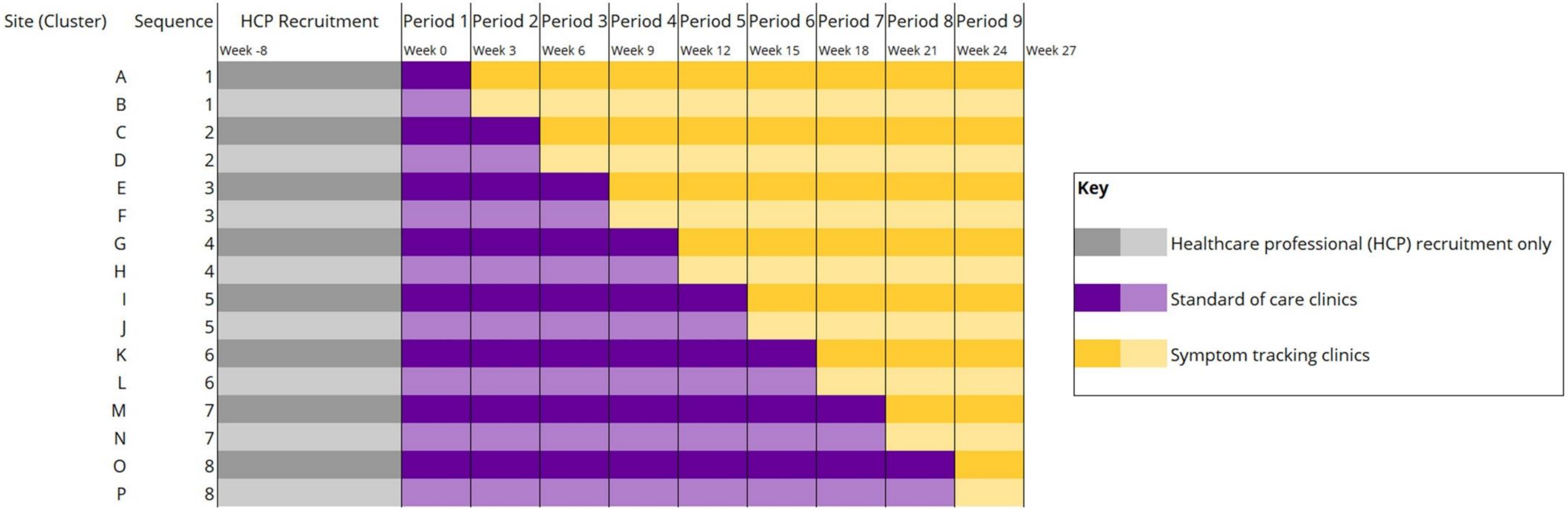

Figure S2 – Screenshots from the REMORA app of the onboarding process and example daily, weekly, and monthly questionnaires

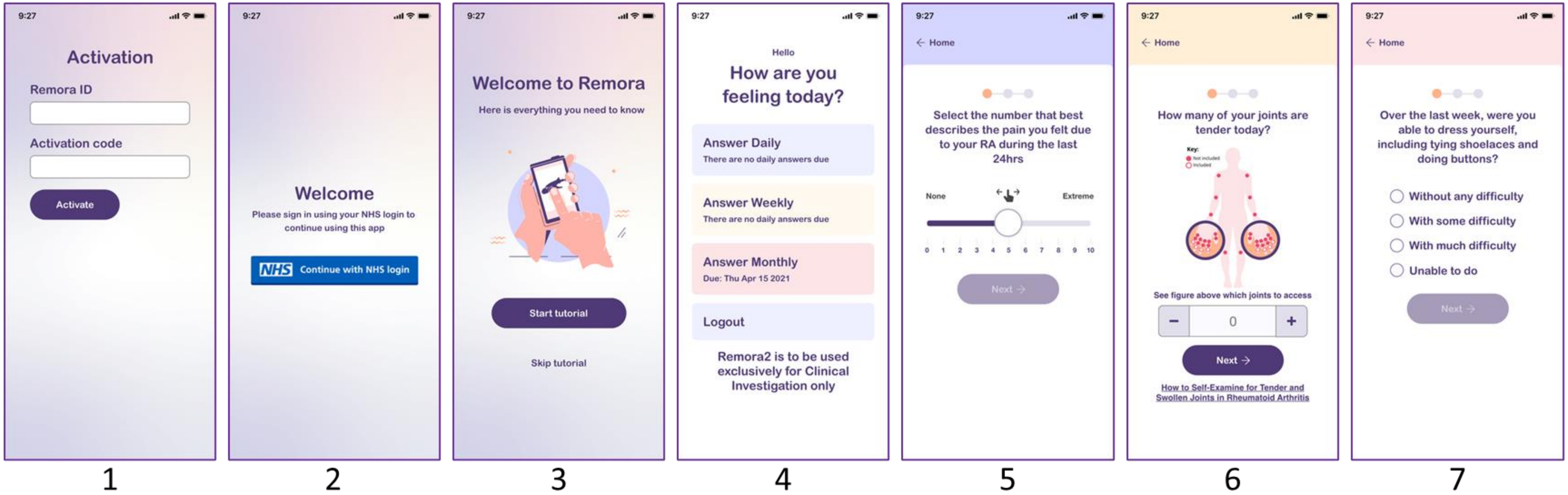

1. Activation page for participants to enter their REMORA ID (unique participant identifier) and study activation code (provided in the welcome email)
2. NHS login page for participants to link an existing NHS login account with the app, or create and link a new account
3. Welcome message displayed to participants who have successfully registered and linked NHS Login account
4. App home-screen image
5. Example daily question (pain score from Rheumatoid Arthritis Impact of Disease score (RAID))
6. Example weekly question (tender joint count)
7. Example monthly question (item from Stanford Health Assessment Questionnaire (HAQ))

Figure S3 – The REMORA interactive dashboard to view people's symptom scores within the participant's electronic health record

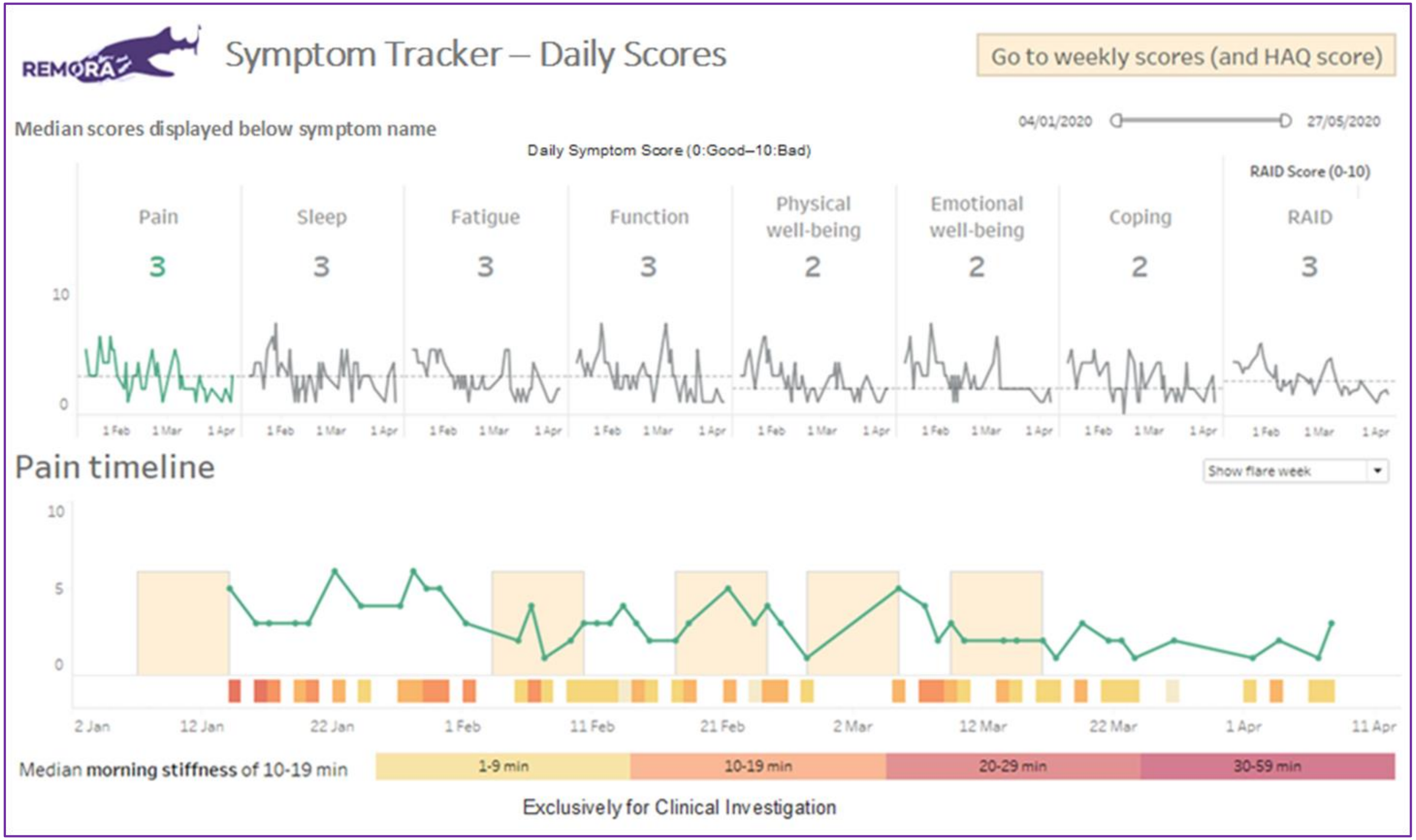

Table S1. Completed CONSORT 2010 checklist extended for pilot or feasibility trials. Please note that some items are completed as n/a (not applicable) because allocation was not randomised

Section/Topic	Item No	Checklist item	Reported on page No
**Title and abstract**			
	1a	Identification as a pilot or feasibility randomised trial in the title	1
	1b	Structured summary of pilot trial design, methods, results, and conclusions (for specific guidance see CONSORT abstract extension for pilot trials)	3
**Introduction**			
Background and objectives	2a	Scientific background and explanation of rationale for future definitive trial, and reasons for randomised pilot trial	4
	2b	Specific objectives or research questions for pilot trial	4
**Methods**			
Trial design	3a	Description of pilot trial design (such as parallel, factorial) including allocation ratio	n/a
	3b	Important changes to methods after pilot trial commencement (such as eligibility criteria), with reasons	n/a
Participants	4a	Eligibility criteria for participants	5-6; 10
	4b	Settings and locations where the data were collected	8-10
	4c	How participants were identified and consented	5-6; 10
Interventions	5	The interventions for each group with sufficient details to allow replication, including how and when they were actually administered	6; Fig S2 and S3
Outcomes	6a	Completely defined prespecified assessments or measurements to address each pilot trial objective specified in 2b, including how and when they were assessed	7-8
	6b	Any changes to pilot trial assessments or measurements after the pilot trial commenced, with reasons	n/a
	6c	If applicable, prespecified criteria used to judge whether, or how, to proceed with future definitive trial	Table 1
Sample size	7a	Rationale for numbers in the pilot trial	7
	7b	When applicable, explanation of any interim analyses and stopping guidelines	n/a
Randomisation:			
Sequence generation	8a	Method used to generate the random allocation sequence	n/a
	8b	Type of randomisation(s); details of any restriction (such as blocking and block size)	n/a
Allocation concealment mechanism	9	Mechanism used to implement the random allocation sequence (such as sequentially numbered containers), describing any steps taken to conceal the sequence until interventions were assigned	n/a
Implementation	10	Who generated the random allocation sequence, who enrolled participants, and who assigned participants to interventions	n/a
Blinding	11a	If done, who was blinded after assignment to interventions (for example, participants, care providers, those assessing outcomes) and how	n/a
	11b	If relevant, description of the similarity of interventions	n/a
Statistical methods	12	Methods used to address each pilot trial objective whether qualitative or quantitative	10
**Results**			

Participant flow (a diagram is strongly recommended)	13a	For each group, the numbers of participants who were approached and/or assessed for eligibility, randomly assigned, received intended treatment, and were assessed for each objective	Figures 2-3; 11-13
	13b	For each group, losses and exclusions after randomisation, together with reasons	Figures 2-3
Recruitment	14a	Dates defining the periods of recruitment and follow-up	Figure 1
	14b	Why the pilot trial ended or was stopped	n/a
Baseline data	15	A table showing baseline demographic and clinical characteristics for each group	Tables 3 and S3
Numbers analysed	16	For each objective, number of participants (denominator) included in each analysis. If relevant, these numbers should be by randomised group	11-13
Outcomes and estimation	17	For each objective, results including expressions of uncertainty (such as 95% confidence interval) for any estimates. If relevant, these results should be by randomised group	11-13; Tables 3 and S3
Ancillary analyses	18	Results of any other analyses performed that could be used to inform the future definitive trial	13-14
Harms	19	All important harms or unintended effects in each group (for specific guidance see CONSORT for harms)	None reported
	19a	If relevant, other important unintended consequences	n/a
**Discussion**			
Limitations	20	Pilot trial limitations, addressing sources of potential bias and remaining uncertainty about feasibility	15-16
Generalisability	21	Generalisability (applicability) of pilot trial methods and findings to future definitive trial and other studies	16
Interpretation	22	Interpretation consistent with pilot trial objectives and findings, balancing potential benefits and harms, and considering other relevant evidence	14-16
	22a	Implications for progression from pilot to future definitive trial, including any proposed amendments	16
**Other information**			
Registration	23	Registration number for pilot trial and name of trial registry	3
Protocol	24	Where the pilot trial protocol can be accessed, if available	Not available
Funding	25	Sources of funding and other support (such as supply of drugs), role of funders	17
	26	Ethical approval or approval by research review committee, confirmed with reference number	17

Table S2. Completed consolidated criteria for reporting qualitative studies (COREQ) checklist		
**No. Item**	**Guide questions/description**	**Reported on Page #**
**Domain 1: Research team and reflexivity**		
Personal Characteristics		
1. Inter viewer/facilitator	Which author/s conducted the interview or focus group?	10
2. Credentials	What were the researcher's credentials? E.g. PhD, MD	10
3. Occupation	What was their occupation at the time of the study?	10
4. Gender	Was the researcher male or female?	10
5. Experience and training	What experience or training did the researcher have?	10
Relationship with participants		10
6. Relationship established	Was a relationship established prior to study commencement?	10
7. Participant knowledge of the interviewer	What did the participants know about the researcher? e.g. personal goals, reasons for doing the research	6
8. Interviewer characteristics	What characteristics were reported about the inter viewer/facilitator? e.g. Bias, assumptions, reasons and interests in the research topic	10
**Domain 2: study design**		
Theoretical framework		
9. Methodological orientation and Theory	What methodological orientation was stated to underpin the study? e.g. grounded theory, discourse analysis, ethnography, phenomenology, content analysis	5
Participant selection		
10. Sampling	How were participants selected? e.g. purposive, convenience, consecutive, snowball	6; 10
11. Method of approach	How were participants approached? e.g. face-to-face, telephone, mail, email	5-6
12. Sample size	How many participants were in the study?	13
13. Non-participation	How many people refused to participate or dropped out? Reasons?	6
Setting		
14. Setting of data collection	Where was the data collected? e.g. home, clinic, workplace	10
15. Presence of non-participants	Was anyone else present besides the participants and researchers?	10
16. Description of sample	What are the important characteristics of the sample? e.g. demographic data, date	Table 3; 13
Data collection		
17. Interview guide	Were questions, prompts, guides provided by the authors? Was it pilot tested?	10
18. Repeat interviews	Were repeat interviews carried out? If yes, how many?	10

19. Audio/visual recording	Did the research use audio or visual recording to collect the data?	10
20. Field notes	Were field notes made during and/or after the interview or focus group?	10
21. Duration	What was the duration of the inter views or focus group?	13
22. Data saturation	Was data saturation discussed?	13
23. Transcripts returned	Were transcripts returned to participants for comment and/or correction?	10
**Domain 3: analysis and findings**		
Data analysis		
24. Number of data coders	How many data coders coded the data?	10
25. Description of the coding tree	Did authors provide a description of the coding tree?	Not available
26. Derivation of themes	Were themes identified in advance or derived from the data?	10
27. Software	What software, if applicable, was used to manage the data?	10
28. Participant checking	Did participants provide feedback on the findings?	10
Reporting		
29. Quotations presented	Were participant quotations presented to illustrate the themes/findings? Was each quotation identified? e.g. participant number	Table 4
30. Data and findings consistent	Was there consistency between the data presented and the findings?	Table 4; 13-14
31. Clarity of major themes	Were major themes clearly presented in the findings?	Table 4; 13-14
32. Clarity of minor themes	Is there a description of diverse cases or discussion of minor themes?	Table 4; 13-14

Table S3. Comparisons of demographic characteristics for a) those who were recruited vs. those who on-boarded and b) low vs high adherers*.

		Recruited vs on-boarded participants		***Low vs High adherers***	
		Recruited (n=52)	*On-boarded* (n=32)	*Low* (n=6)	*High* (n=20)
Female (n, (%) (95% CI))		36 (69.2) (54.9-81.3)	23 (71.9) (53.3-86.3)	6 (100) (54.1-100)	13 (65.0) (40.8-84.6)
Age (median (IQR))		58.0 (48.0-65.3)	56.5 (48.0-63.0)	41.5 (34.5-55.3)	61.0 (50.5-65.3)
Ethnicity (n, (%) (95% CI))	White	45 (86.5) (74.2-94.4)	30 (93.8) (79.2-99.2)	4 (66.7) (22.3-95.7)	20 (100) (83.2-100)
Smoking[1] (n, (%) (95% CI))	Current	11 (21.2) (11.1-34.7)	5 (15.7) (5.3-32.8)	1 (16.7) (0.4-64.1)	3 (15.0) (3.2-37.9)
	Former	22 (42.3) (28.7-56.8)	13 (40.6) (23.7-59.3)	2 (33.3) (4.3-77.7)	9 (45.0) (23.1-68.5)
	Never	18 (34.6) (22.0-49.0)	13 (40.6) (23.7-59.3)	3 (50.0) (11.8-88.2)	8 (40.0) (19.1-64.0)
BMI[2] (n, (%) (95% CI))	Normal	17 (32.7) (20.3-47.1)	11 (34.4) (18.6-53.2)	2 (33.3) (4.3-77.7)	5 (25.0) (8.7-49.1)
	Over	19 (36.5) (23.6-51.0)	10 (31.2) (16.1-50.0)	1 (16.7) (0.4-64.1)	9 (45.0) (23.1-68.5)
	Obese	16 (30.8) (18.7-45.1)	11 (34.4) (18.6-53.2)	3 (50.0) (11.8-88.2)	6 (30.0) (11.9-54.3)
Diagnosis[3] (n, (%) (95% CI))	Rheumatoid arthritis	50 (96.2) (86.8-99.5)	31 (96.9) (83.8-99.9)	6 (100) (54.1-100)	20 (100) (83.2-100)
	Suspected rheumatoid arthritis	1 (1.9) (0.1-10.2)	1 (3.1) (0.1-16.2)	0 (0-45.9)	0 (0-16.8)
	Undifferentiated IA	1 (1.9) (0.1-10.2)	0 (0-10.9)	0 (0-45.9)	0 (0-16.8)
Disease duration (median (IQR))		3 (0-10.3)	3.5 (0-10.8)	1.0 (0-5.8)	3.0 (0.8-10.8)

Abbreviations: CI, confidence interval; IA, inflammatory arthritis; IQR, interquartile range
[1]smoking status missing from medical record for one on-boarded participant; [2]no one is underweight so category omitted; [3]no one suspected IA so category omitted; Low adherers– symptoms reported on <25% days; High adherers - symptoms reported on >60% days.*
